# Micheliolide exerts effects in myeloproliferative neoplasms through inhibiting STAT3/5 phosphorylation via covalent binding to STAT3/5 proteins

**DOI:** 10.1097/BS9.0000000000000168

**Published:** 2023-07-12

**Authors:** Huijun Huang, Jinqin Liu, Lin Yang, Yiru Yan, Meng Chen, Bing Li, Zefeng Xu, Tiejun Qin, Shiqiang Qu, Liang Wang, Gang Huang, Yue Chen, Zhijian Xiao

**Affiliations:** a State Key Laboratory of Experimental Hematology, National Clinical Research Center for Blood Diseases, Haihe Laboratory of Cell Ecosystem, Institute of Hematology & Blood Diseases Hospital, Chinese Academy of Medical Sciences & Peking Union Medical College, Tianjin 300020, China; b MDS and MPN Centre, Institute of Hematology and Blood Diseases Hospital, Chinese Academy of Medical Sciences & Peking Union Medical College, Tianjin, China; c State Key Laboratory of Medicinal Chemical Biology, College of Chemistry, Nankai University, 94 Weijin Road, Tianjin, China; d Department of Cell System & Anatomy, the University of Texas Health Science Center at San Antonio, San Antonio, TX 78229, USA; e Mays Cancer Center, Joe R. & Teresa Lozano Long School of Medicine, the University of Texas Health Science Center at San Antonio, San Antonio, TX 78229, USA; f Hematologic Pathology Center, Institute of Hematology and Blood Diseases Hospital, Chinese Academy of Medical Sciences & Peking Union Medical College, Tianjin, China.

**Keywords:** Micheliolide, Myeloproliferative neoplasms, Ruxolitinib, STAT3/5, Suboptimal response

## Abstract

Ruxolitinib is a cornerstone of management for some subsets of myeloproliferative neoplasms (MPNs); however, a considerable number of patients respond suboptimally. Here, we evaluated the efficacy of micheliolide (MCL), a natural guaianolide sesquiterpene lactone, alone or in combination with ruxolitinib in samples from patients with MPNs, *JAK2*V617F-mutated MPN cell lines, and a *Jak2*V617F knock-in mouse model. MCL effectively suppressed colony formation of hematopoietic progenitors in samples from patients with MPNs and inhibited cell growth and survival of MPN cell lines in vitro. Co-treatment with MCL and ruxolitinib resulted in greater inhibitory effects compared with treatment with ruxolitinib alone. Moreover, dimethylaminomicheliolide (DMAMCL), an orally available derivative of MCL, significantly increased the efficacy of ruxolitinib in reducing splenomegaly and cytokine production in *Jak2*V617F knock-in mice without evident effects on normal hematopoiesis. Importantly, MCL could target the *Jak2*V617F clone and reduce mutant allele burden in vivo. Mechanistically, MCL can form a stable covalent bond with cysteine residues of STAT3/5 to suppress their phosphorylation, thus inhibiting JAK/STAT signaling. Overall, these findings suggest that MCL is a promising drug in combination with ruxolitinib in the setting of suboptimal response to ruxolitinib.

## 1. INTRODUCTION

Myeloproliferative neoplasms (MPNs) are clonal hematologic stem cell disorders characterized by excessive output of myeloid cells and an inherent risk for leukemic transformation. Polycythemia vera (PV), essential thrombocythemia (ET), and primary myelofibrosis (PMF) constitute classic *BCR-ABL* negative MPNs.^1^ These MPN subsets share constitutively activated Janus kinase (JAK)/signal transducer and activator of transcription (STAT) signaling, usually driven by somatic mutations in *JAK2*, myeloproliferative leukemia protein (*MPL*), or calreticulin (*CALR*); accordingly, these variants are referred to as “MPN driver mutations.”^2^ The most common driver mutation in MPNs is a V617F point mutation in *JAK2*, which occurs in more than 95% of patients with PV and approximately 50% of patients with ET or PMF.^3^

Given the central role of aberrant JAK/STAT signaling in MPN pathobiology, JAK inhibitors represent a standard treatment for certain subsets of MPNs. Ruxolitinib, a JAK1/2 inhibitor, has been approved for the treatment of patients with intermediate- and high-risk MF, and as a second-line therapy for PV.^4^ Although ruxolitinib can provide some clinical benefits, including superior reduction of splenomegaly and symptom improvement,^5,6^ it has several drawbacks. First, dose-dependent adverse effects, especially hematological toxicities, limit the application of ruxolitinib in a substantial proportion of patients.^7^ Second, approximately half of patients treated with ruxolitinib reportedly discontinued treatment after a 3-year period, a third of whom reported suboptimal responses (defined as a lack or loss of spleen response).^8^ Moreover, ruxolitinib has extremely limited effects on eradicating malignant clones, meaning the molecular response it elicits is modest.^9^ Several new drugs have been developed to overcome the limitations of ruxolitinib. For example, the novel JAK inhibitors pacritinib and momelotinib have proven to be effective, particularly for patients with thrombocytopenia and anemia, respectively.^10,11^ In addition, numerous clinical trials evaluating agents that rationally hold potential for synergism/additivity with ruxolitinib, such as PI3K/AKT/mTOR and Bcl-2/Bcl-xL inhibitors, are ongoing.^12,13^

Micheliolide (MCL), a natural guaianolide sesquiterpene lactone discovered in *Michelia compressa*, exhibits strong therapeutic efficacy towards various inflammatory diseases and neoplasms through multiple bioactivities, mainly inhibition of the nuclear factor κB (NF-κB) pathway and promotion of reactive oxygen species.^14–16^ Its water-soluble Michael adduct, dimethylaminomicheliolide (DMAMCL), displays excellent plasma stability and efficacy in vivo.^17^ Importantly, MCL was found to selectively eradicate CD34^+^CD38^-^ leukemic stem cells in acute myeloid leukemia.^18^ Therefore, we hypothesized that MCL could also exert antineoplastic and anti-inflammatory activities in MPNs and might have synergistic or additive effects with ruxolitinib, and potentially contribute to targeting of malignant clones.

Here, we evaluated the efficacy of MCL/DMAMCL in bone marrow (BM) cells of patients with MPNs, *JAK2*V617F-positive MPN cell lines, and murine models, both alone and in combination with ruxolitinib. We found that MCL significantly inhibits the growth of MPN cells in vitro, mainly by covalently binding to STAT3/5 proteins and inhibiting their activation. Moreover, DMAMCL significantly improved the efficacies of ruxolitinib in reducing splenomegaly and cytokine production in *Jak2*V617F mice; importantly, it also reduced mutant allele burden in vivo. Effective doses of DMAMCL did not obviously affect the normal hematopoiesis of wild-type (WT) mice. Collectively, this study provides evidence for combining MCL and ruxolitinib in patients with MPNs who respond suboptimally to ruxolitinib.

## 2. MATERIALS AND METHODS

### 2.1. Patient samples

BM samples and clinical data from patients diagnosed with MPNs according to 2016 World Health Organization (WHO) MPN definitions^19^ were obtained from the MDS and MPN Center at Blood Disease Hospital. Clinical and molecular characteristics of MPN patients are listed in Supplemental Table 1, http://links.lww.com/BS/A68. All patients signed an informed consent compliant with the Declaration of Helsinki. The study protocol involving in human samples was approved by the Ethics Committee of Blood Disease Hospital (approval no.: KT2015010-EC-2).

### 2.2. Drugs, cell lines, and cell culture

MCL and DMAMCL were kindly provided by Dr. Yue Chen (Nankai University, Tianjin, China). Ruxolitinib was purchased from MedChemExpress (Monmouth Junction, New Jersey). MCL and ruxolitinib were dissolved in dimethyl sulfoxide (DMSO; stock solutions of 40 and 10 mM, respectively) and further diluted in culture medium. DMAMCL was dissolved in phosphate-buffered saline (PBS) for in vivo use. *JAK2*V617F mutant UKE1 and SET2 human cell lines, as well as murine Ba/F3 and Ba/F3-EPOR-JAK2V617F cell lines, were kind gifts from Dr. Gang Huang (University of Texas Health Science Center at San Antonio, Texas). UKE1 cells were cultured in Iscove’s Modified Dulbecco’s Medium supplemented with 10% horse serum, 10% fetal bovine serum (FBS), 1 μM hydrocortisone and penicillin/streptomycin. SET2 cells were cultured in Roswell Park Memorial Institute (RPMI) 1640 medium supplemented with 20% FBS and penicillin/streptomycin. Ba/F3 and Ba/F3-EPOR-JAK2V617F cells were maintained in RPMI 1640 supplemented with 10% FBS, 10 ng/mL IL-3, and penicillin/streptomycin. Logarithmically growing cells were exposed to the indicated concentrations of MCL and/or ruxolitinib before functional studies.

### 2.3. Mice

Cre-inducible *Jak2*^*V617F/+*^ mice and hematopoietic specific *Vav1*-Cre transgenic mice were used as previously described.^20^ Reporter tdTomato mice under a CAG promoter were obtained from Cyagen Biosciences (Santa Clara, California). For in vivo drug studies, 6- to 8-week-old CD45.1 C57BL/6 female recipient mice were transplanted with 3 × 10^6^ total BM nucleated cells (BMNCs) from *Jak2*^*V617F/+*^*/Vav1*-Cre (*Jak2*^*VF*^) CD45.2 donor mice and then randomized into treatment groups 8 weeks post-transplantation according to blood counts. Next, mice were treated with vehicle (PBS), DMAMCL (70 mg/kg/d), ruxolitinib (60 mg/kg/d), or combined therapy by oral gavage for 4 weeks. Coeval WT C57BL/6 mice were randomized to receive vehicle or DMAMCL (70 mg/kg/d) treatments to assess drug toxicity. The dosage of DMAMCL was selected according to previous research.^21^ To generate a chimeric mouse model, a mixture of BMNCs from *Jak2*^*V617F/+*^/tdTomato/*Vav1*-Cre (*Jak2* ^*VF*^*/td*) mice and BMNCs from WT littermates were engrafted into lethally irradiated recipients. The treatment regimen for chimeric mice was as described above. Mice were housed in the animal barrier facility at State Key Laboratory of Experimental Hematology. All animal experimental protocols were approved by the Institutional Animal Care and Use Committee of State Key Laboratory of Experimental Hematology (approval no.: KT2015010-EC-2). Details of animal experiments are contained in the Supplementary Methods http://links.lww.com/BS/A68.

### 2.4. Cell viability, apoptosis, and cell cycle assays

Cells were seeded into 96-well plates at a density of 1 × 10^4^ cells/well with increasing concentrations of MCL and/or ruxolitinib, in triplicate. After 24, 48, or 72 hours of treatment, cell viability (normalized to wells containing an equivalent volume of DMSO) was measured using a cell counting kit-8 (CCK-8) assay (Dojindo Laboratories, Kumamoto, Japan). Synergy analysis was performed using CompuSyn software v.1.0 (ComboSyn, Inc.) based on the Chou-Talalay method. CI values of <1, =1, and >1 indicate synergistic, additive, and antagonistic effects, respectively.

Percentages of apoptotic cells after 48 hours of drug exposure were analyzed using a PE Annexin V Apoptosis Detection Kit (BD Biosciences, Franklin Lakes, New Jersey) according to the manufacturer’s protocol. Cell cycle distribution after 24 hours of MCL treatment was analyzed by propidium iodide (PI) staining (Beyotime, Shanghai, China). Fluorescence was measured with a FACS Canto II flow cytometer (BD Biosciences) and data were analyzed with FlowJo software v.10 (RRID:SCR_008520).

### 2.5. Colony-forming unit assay

For primary samples collected from patients with MPNs, BM mononuclear cells (BMMNCs) were sorted and seeded at a density of 5 × 10^4^/mL in triplicate in methylcellulose medium (MethoCult H4435; Stem Cell Technologies, Vancouver, Canada) supplemented with MCL and/or ruxolitinib. After incubation for 14 days, numbers of burst-forming unit-erythroid (BFU-E); colony-forming unit (CFU)-granulocyte, macrophage (CFU-GM); and CFU-granulocyte, erythrocyte, macrophage, megakaryocyte (CFU-GEMM) were counted. UKE1 cells (2 × 10^3^/mL) pretreated with MCL and/or ruxolitinib for 24 hours were plated in triplicate in medium (MethoCult H4230). Numbers of colonies were counted after 14 days. Murine BM cells from *Jak2* ^*VF*^*/td* or *Jak2*^*WT*^ littermate mice were mixed at a 50% ratio and then seeded at a density of 5 × 10^4^/mL in triplicate in medium (MethoCult M3434) supplemented with MCL and/or ruxolitinib. Proportions of mutant colonies were calculated after 7 days of culture.

### 2.6. Western blot analysis

After treatment, cells were collected in lysis buffer supplemented with Phosphatase Inhibitor Cocktail II (Calbiochem, San Diego, California). Next, whole-cell lysates were boiled in SDS Loading Buffer (Beyotime). Total protein was separated by polyacrylamide gel electrophoresis (Yeasen, Shanghai, China). Primary antibodies used for western blot (WB) include: Bcl-xL [RRID: AB_2228008; Cat. No. 2764; Cell Signaling Technology (CST), Danvers, Massachusetts], Bcl-2 (RRID: AB_1903909; Cat. No. 4223, CST), caspase 9 (RRID: AB_2068620; Cat. No. 9508, CST), caspase 3 (RRID: AB_2798429; Cat. No, 14220, CST), poly(ADP-ribose) polymerase (PARP) (RRID: AB_659884; Cat. No. 9532, CST), STAT3 (RRID: AB_331269; Cat. No. 4904, CST), phosphorylated (p-)STAT3 (Tyr705; RRID: AB_2491009; Cat. No. 9145, CST), STAT5 (RRID: AB_2737403; Cat. No. 94205, CST), p-STAT5 (Tyr694; RRID: AB_10544692; Cat. No. 4322, CST), IκBα (RRID: AB_390781; Cat. No. 4814, CST), p-IκBα (Ser32/36; RRID: AB_2267145; Cat. No. 9246, CST), IKKβ (RRID: AB_11024092; Cat. No. 8943, CST), p-IKKα/β (Ser176/180; RRID: AB_2079382; Cat. No. 2697, CST), NF-κB p65 (RRID: AB_10859369; Cat. No. 8242, CST), p-NF-κB p65 (Ser536; RRID: AB_331284; Cat. No. 3033, CST), eIF2α (RRID: AB_10692650; Cat. No. 5324, CST), p-eIF2α (Ser51; RRID: AB_2096481; Cat. No. 3398, CST), β-actin (RRID: AB_10950489; Cat. No. 8457, CST), and glyceraldehyde 3-phosphate dehydrogenase (GAPDH) (RRID: AB_2885058; Cat. No. BM1623, BOSTER Bio, Wuhan, China).

### 2.7. Molecular docking

Three-dimensional (3D) structures of STAT3 and STAT5B were obtained from Protein Data Bank with codes 6TLC and 6MBW, respectively. The STAT5A structure was predicted using a homology modeling method^22^ according to STAT5B, as no crystal structure of STAT5A was previously reported. The molecular structure of MCL was taken from PubChem and optimized with the MOPAC program.^23^ The initial structures of all proteins and MCL were processed with AutoDock Tools 1.5.6. Molecular docking was realized using the AutoDock 4.2.6 software package (RRID:SCR_012746). Details are contained in the Supplementary Methods, http://links.lww.com/BS/A68.

### 2.8. Biotin-based pull-down assay

Biotinylated MCL (biotin-MCL) and its inactive analogue S-MCL (biotin-S-MCL) were kindly provided by Dr. Yue Chen (Nankai University, Tianjin, China).^24^ Cell lysates from UKE1 and SET2 cells were incubated with 20 μM of biotin-MCL or biotin-S-MCL at room temperature (RT) for 1 hour. After incubation, prewashed streptavidin agarose beads (Thermo Fisher Scientific, Waltham, Massachusetts) were added to each sample and incubated at RT for 1 hour, followed by PBS (containing 0.5% sodium dodecyl sulfate) washing for four times to remove non-specific bound proteins. Next, bead-bound proteins were eluted with violent shaking and boiled in SDS Loading Buffer. The pull-down protein was identified by WB with primary antibodies against STAT3 and STAT5.

### 2.9. Statistical analysis

Results are shown as mean ± standard deviation (SD) or standard error of the mean (SEM), as indicated. Statistical significance was determined by one-way analysis of variance (ANOVA) or unpaired Student’s t-test using GraphPad Prism software (v.7, RRID: SCR_002798). *P* values < .05 are considered statistically significant.

## 3. RESULTS

### 3.1. MCL alone or combined with ruxolitinib inhibits colony formation of hematopoietic stem/progenitor cells from patients with MPNs

**Figure 1.**
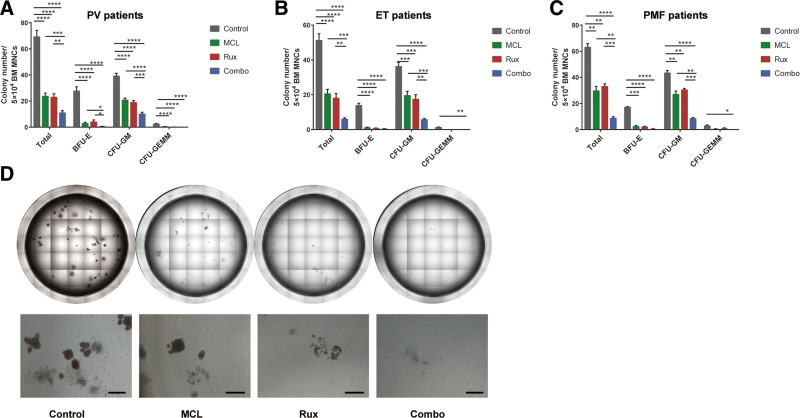
MCL alone or combined with ruxolitinib inhibits colony formation of hematopoietic stem/progenitor cells from patients with MPNs. (A–C) BMMNCs were isolated from four individuals with PV (A), two individuals with ET (B), and one individual with PMF (C) and seeded in methylcellulose in triplicate with MCL (5 μM) and ruxolitinib (50 nM) alone or in combination. Numbers of total colonies, BFU-E, CFU-GM, and CFU-GEMM were counted 14 days later. (D) Representative panoramic images captured by a high-content analysis system (PerkinElmer) with a magnification of 10× for each well (upper) and representative images captured by inverted microscope (Nikon) with an original magnification of 4× (lower) of colony formation after 14 days of culture with MCL and/or ruxolitinib. Scale bar, 500 μm. Data are presented as the mean ± SEM. * *P* < .05, ** *P* < .01, *** *P* < .001, **** *P* < .0001. BFU-E = burst-forming unit-erythroid, BMMNC = Bone marrow mononuclear cell, CFU-GM = colony-forming unit-granulocyte macrophage, CFU-GEMM = colony-forming unit-granulocyte, erythrocyte, macrophage, megakaryocyte, ET = essential thrombocythemia, MCL = micheliolide, PMF = primary myelofibrosis, PV = polycythemia vera.

To explore the therapeutic potential of MCL in human MPN settings, we first assessed the effect of MCL and/or ruxolitinib on the in vitro clonogenic ability of primary cells derived from four individuals with PV, two individuals with ET, and one individual with PMF. After 14 days of co-culture, regardless of the diagnosis subtypes and driver mutations of patients, MCL alone significantly decreased total colony numbers, as well as BFU-E and CFU-GM numbers (*P* < .01, Fig. 1A–C), especially inhibited BFU-E formation, as reflected by lower numbers and smaller areas per colony (Fig. 1D). The Combination of MCL and ruxolitinib was superior in terms of reducing BFU-E and CFU-GM numbers in samples from patients with PV (*P* < .05), and CFU-GM numbers in samples of patients with ET or PMF (*P* < .01) compared with each single agent (Fig. 1A–C). These results preliminarily suggest that MCL has an inhibitory effect on MPN patient-derived cells and can enhance the effect of ruxolitinib.

### 3.2. MCL alone or in combination with ruxolitinib reduces cell viability and induces apoptosis in *JAK2*V617F-mutated cell lines

**Figure 2.**
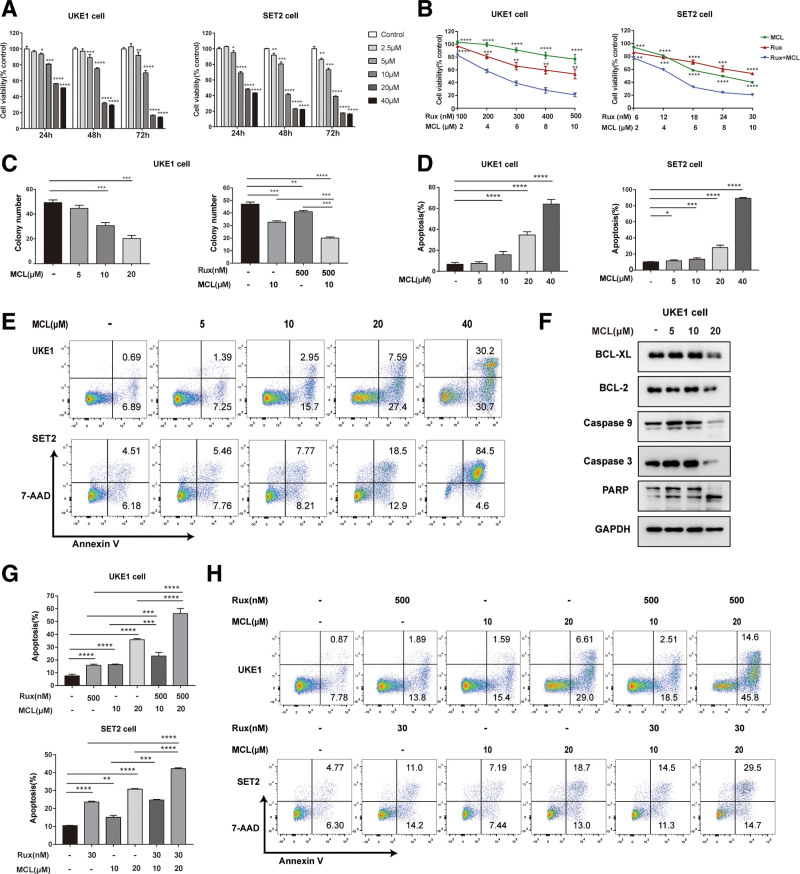
MCL inhibits cell viability and induces cell apoptosis of *JAK2*V617F mutant cells. (A) UKE1 and SET2 cells were treated with dose-escalated MCL (5–40 μM) for 24, 48, or 72 h, and then an assessment for cell viability using CCK-8 assays. (B) UKE1 and SET2 cells were treated with the indicated concentrations of MCL and ruxolitinib alone or in combination for 72 h before assessing cell viabilities. Rux: ruxolitinib. Viability experiments were performed in triplicate. (C) UKE1 cells were pretreated with the indicated concentrations of MCL alone or combined with ruxolitinib (500 nM) for 24 h, then collected and seeded in methylcellulose medium in triplicate. Colony numbers were determined on day 14. (D and E) UKE1 and SET2 cells were treated with dose-escalated MCL (5–40 μM) for 48 h, then subjected apoptosis detection with a 7-AAD/Annexin V double staining assay (n = 3 independent experiments). (F) Western blot (WB) analysis results are shown for BCL-XL, BCL-2, caspase 9, caspase 3, and PARP proteins from UKE1 cells treated with MCL (5–20 μM) for 24 h. (G and H) UKE1 and SET2 cells were treated with MCL (10, 20 μM) alone or in combination with ruxolitinib for 48 h, and then apoptosis was detected (n = 3). Data are presented as the mean ± SD. *P < .05, **P < .01, ***P < .001, ****P < .0001. CCK-8 = cell counting kit-8, MCL = micheliolide.

We next evaluated the effect of MCL on cell viability in MPN cell lines expressing the *JAK2*V617F mutation. Treatment with MCL at or above 5 μM significantly reduced the viability of UKE1 and SET2 cells in a dose- and time-dependent manner (Fig. 2A). Next, to determine whether MCL could enhance ruxolitinib-driven growth suppression, we treated UKE1 and SET2 cells with specific concentrations of MCL and ruxolitinib [20%, 40%, 60%, 80%, and 100% of the approximate half-maximal inhibitory concentration value (listed in Supplementary Table 2, http://links.lww.com/BS/A68)] alone or in combination for 72 hours. As shown in Figure 2B, combined treatments all resulted in greater inhibition of UKE1 and SET2 cell viability compared with either single agent (all *P* < .01). Accordingly, the combination index (CI) was calculated to assess potential synergy. We observed no obvious synergism but there was an additive effect between MCL and ruxolitinib, given that CI values for different combinations were all close to 1 (Supplementary Table 3, http://links.lww.com/BS/A68). Moreover, pre-exposure to MCL (at or above 10 μM) significantly reduced the colony-forming capacity of UKE1 cells (*P* < .001), which enhanced the effect of ruxolitinib alone (*P* < .001, Fig. 2C).

To understand how MCL causes proliferation arrest, we first assessed cell cycle progression. We found no obvious cell cycle arrest upon MCL treatment (Supplementary Figure 1, http://links.lww.com/BS/A69). Next, we examined cell apoptosis by performing flow cytometry. Treatment with MCL dose-dependently induced apoptosis of UKE1 and SET2 cells (Fig. 2D and E); similar effects were observed for murine Ba/F3-EPOR-*JAK2*V617F cell lines (Supplementary Figure 2, http://links.lww.com/BS/A70). More importantly, compared with its effects on Ba/F3-EPOR-*JAK2*V617F cells, MCL increased apoptosis of their WT counterparts at significantly higher concentrations (*P* < .01, Supplementary Figure 2, http://links.lww.com/BS/A70), indicating that MCL has relatively greater selectivity towards *JAK2*V617F-mutated cells. WB analysis confirmed that MCL was able to reduce levels of anti-apoptotic proteins, such as Bcl-xL and Bcl-2, and induce cleavage of caspase 3, caspase 9, and PARP (Fig. 2F), indicating apoptotic pathway activation.^25^ Co-treatment with MCL and ruxolitinib significantly increased apoptosis of UKE1 and SET2 cells compared with treatment with ruxolitinib alone (*P* < .001, Fig. 2G and H). Taken together, these results show that MCL can inhibit survival of *JAK2*V617F-mutated MPN cell lines, alone or in combination with ruxolitinib.

### 3.3. DMAMCL enhances the therapeutic efficacy of ruxolitinib in a *Jak2* V617F mouse model

**Figure 3.**
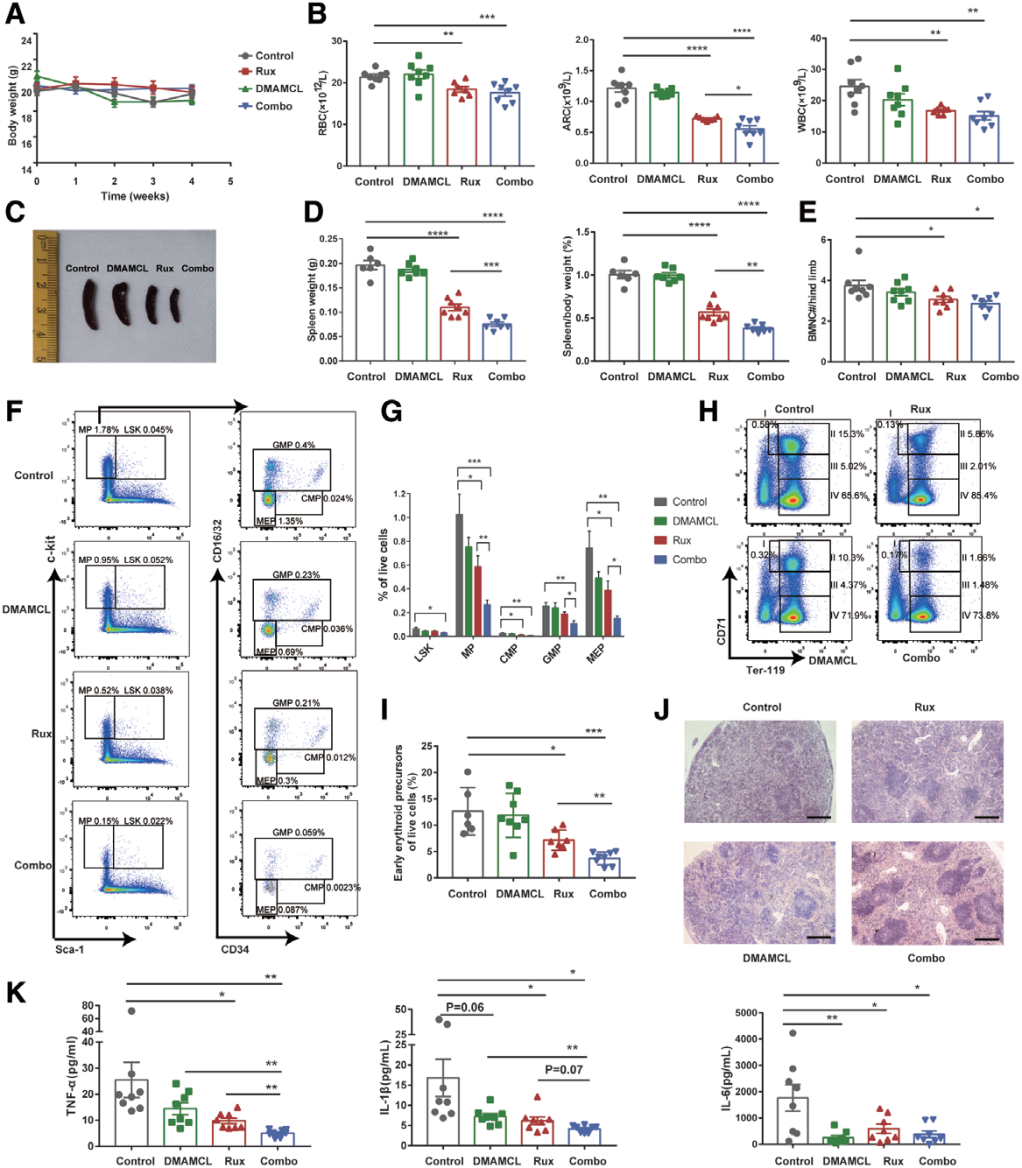
DMAMCL enhances the therapeutic efficacy of ruxolitinib in a *Jak2*V617F mouse model. (A) *Jak2*^*VF*^ recipient mice receiving different treatments were weighed at 0, 1, 2, 3, and 4 weeks after commencing administration (n = 7–8 per group). Combo: combination treatment. (B) RBC, ARC, and WBC counts in PB of recipient mice were assessed after 4 weeks of DMAMCL and/or ruxolitinib treatment (n = 7–8 per group). (C) Representative images of spleens from recipient mice after 4 weeks of administration of different treatments. (D) Spleen weight (left) and percentage of body weight (right) of recipient mice after 4 weeks of DMAMCL and/or ruxolitinib treatment (n = 6–8 per group). (E) BMNC counts per hind limb of recipient mice after treatment with DMAMCL and/or ruxolitinib for 4 weeks (n = 7–8 per group). (F and G) Representative flow cytometric plots (F) and proportions (G) of LSK cells, MPs, CMPs, GMPs, and MEPs in spleens of recipient mice after 4 weeks of DMAMCL and/or ruxolitinib treatment (n = 6–8 per group). (H and I) Representative flow cytometric plots of erythroid precursors (H) and proportions of early erythroid precursors (calculated as I+II) (I) in spleens of recipient mice after 4 weeks of DMAMCL and/or ruxolitinib treatment (n = 6–8 per group). (J) Representative images of H&E staining in spleen biopsy specimens of mice following different treatments for 4 weeks. Original magnification 4×; scale bar 500 μm. (K) Serum inflammatory cytokine levels (TNF-α, IL-1β, and IL-6) of mice were assessed after 4 weeks of administration of different treatments (n = 8 per group). Data are presented as the mean ± SEM. *P < .05, **P < .01, ***P < .001, ****P < .0001. ARC = absolute reticulocyte count, BMNC = Bone marrow nucleated cell, CMP = common myeloid progenitor, DMAMCL = dimethylaminomicheliolide, GMP = granulocyte/macrophage progenitor, LSK = Lin^-^Sca1^+^c-kit^+^, MEP = megakaryocyte/erythroid progenitor, MP = myeloid progenitor, RBC = Red blood cell, WBC = white blood cell.

To further explore the therapeutic potency of DMAMCL as a single agent or in combination with ruxolitinib in vivo, recipients transplanted with BM cells from *Jak2*^*V617F/+*^*/Vav1*-Cre (hereafter *Jak2*^*VF*^) mice were used; in addition, coeval WT mice were employed to evaluate drug toxicity to normal hematopoiesis. Eight weeks after transplantation, all *Jak2*^*VF*^ recipients that developed an MPN phenotype with erythrocytosis^26^ were randomized into four groups that received PBS (Control), DMAMCL, ruxolitinib, or combination therapy for 4 weeks. There was no significant difference in the body weights of *Jak2*^*VF*^ mice receiving any treatment during administration (Fig. 3A). Similarly, no changes in body weight or the weight and histomorphology of liver and spleen of WT mice was observed after 4 weeks of DMAMCL therapy (Supplementary Figure 3A–D, http://links.lww.com/BS/A71). Moreover, DMAMCL did not affect normal hematopoiesis at the evaluated dosage, as PB cell counts, hematopoietic stem and progenitor cells (HSPCs) and differentiated cells in the BM and spleen of WT mice were unaltered following DMAMCL treatment (Supplementary Figure 3E–G, http://links.lww.com/BS/A71). Altogether, these results indicate that the drug dosage was well tolerated.

After 4 weeks of treatment, DMAMCL alone did not obviously relieve erythrocytosis or leukocytosis. However, the combination DMAMCL with ruxolitinib more notably decreased absolute reticulocyte counts (ARCs) compared with ruxolitinib alone (*P* < .05, Fig. 3B). Spleen size, weight, and its relative percentage of body weight were markedly reduced by ruxolitinib (*P* < .0001), and these reductions were significantly enhanced by combination therapy (*P* < .01, Fig. 3C and D). In addition, ruxolitinib or combination therapy moderately reduced BM cellularity (*P* < .05, Fig. 3E).

Flow cytometry analysis showed no obvious changes in proportions of BM HSPCs, except a mild decline in Lin^-^Sca1^+^c-kit^+^ cells and common myeloid progenitors caused by ruxolitinib or combined therapy (Supplementary Figure 4A and B, http://links.lww.com/BS/A72). However, proportions of HSPCs in murine spleen were considerably reduced by ruxolitinib or combined therapy (Fig. 3F and G). Importantly, we observed a notably enhanced decrease in spleen myeloid progenitors (including granulocyte-macrophage progenitors and megakaryocyte-erythrocyte progenitors) by combining DMAMCL compared with our findings with ruxolitinib monotherapy (*P* < .05, Fig. 3F and G). Likewise, compared with ruxolitinib monotherapy, combined therapy led to a more pronounced reduction in spleen early erythroid precursor cells [CD71^high^Ter119^mid-high^, calculated by I plus II according to previous study^27]^ (*P* < .01, Fig. 3H and I).

Collectively, the results described above suggest that DMAMCL can enhance the efficacy of ruxolitinib to relieve splenomegaly and extramedullary hematopoiesis in *Jak2*^*VF*^ mice. To confirm this finding, we performed histopathologic analysis of spleen. Hematoxylin-eosin staining indicates impairment of the normal splenic architecture of recipients in the control group, as characterized by the spreading of red pulp and reduced white pulp with increased megakaryocytes, indicating myeloid trilineage hyperplasia^28^ (Fig. 3J, Supplementary Figure 4C, http://links.lww.com/BS/A72). Treatment with ruxolitinib or DMAMCL could partially reduce extramedullary hematopoiesis and restore the splenic architecture, whereas combined treatment was able to restore near normalcy (Fig. 3J, Supplementary Figure 4C http://links.lww.com/BS/A72).

Overproduction of inflammatory cytokines is involved in MPN pathogenesis and progression.^29^ Thus, we next evaluated serum cytokine levels of mice receiving different treatments. DMAMCL produced a trend towards reduction of serum TNF-α and IL-1β levels, as well as a sharp reduction in IL-6 levels of recipients (*P* < .01) (Fig. 3K). Combined therapy resulted in greater suppression of cytokine production, with further attenuation of TNF-α and IL-1β (*P* < .01; *P* = .07, Fig. 3K).

### 3.4. MCL treatment inhibits *Jak2*V617F-mutant hematopoietic progenitors

**Figure 4.**
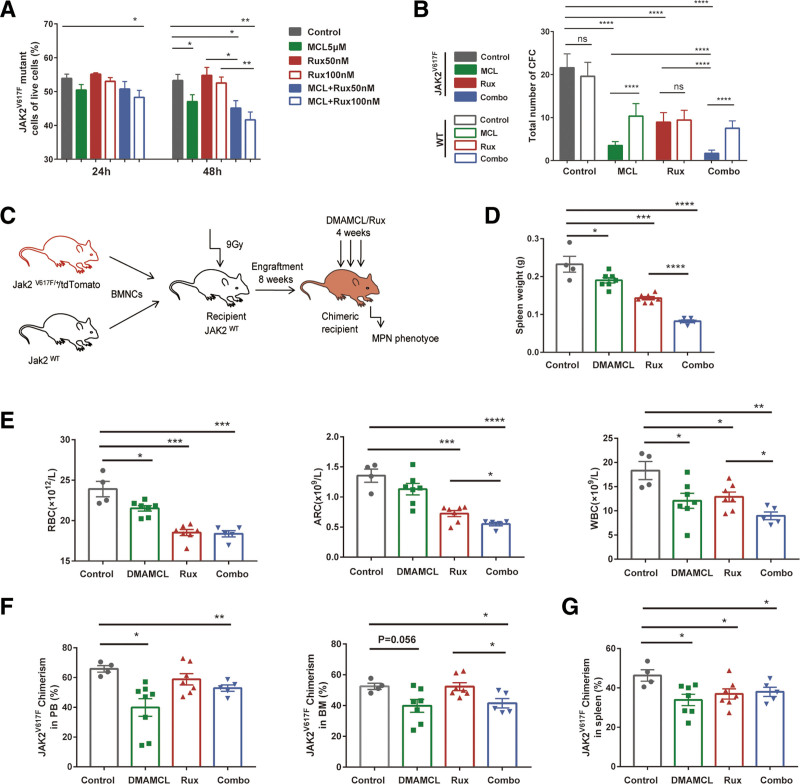
MCL inhibits *Jak2*V617F mutant hematopoietic progenitors. (A) C-kit^+^ cells from *Jak2*^*VF*^*/td* and WT mice were mixed at a 1:1 ratio ex vivo and treated with MCL (5 μM) and ruxolitinib (50, 100 nM) alone or in combination for 24 and 48 h. Proportions of *Jak2*V617F-mutant cells marked with TdTomato fluorescence to total cells were assessed by flow cytometry (n = 4 independent experiments). (B) BMNCs from *Jak2*^*VF*^*/td* and WT mice were mixed at a 1:1 ratio and seeded in methylcellulose with MCL (5 μM) and/or ruxolitinib (50 nM) for 7 d. Subsequently, *Jak2*V617F-mutant colonies were counted using a fluorescence microscope and WT colonies were counted under bright field (n = 4 independent experiments). (C) Schematic representation showing generation of chimeric recipient mice. BMNCs from *Jak2*^*VF*^*/td* and *Jak2*^*WT*^ mice were mixed and injected into lethally irradiated 8-week-old C57BL/6 recipients. (D) Spleens of chimeric recipient mice were weighed after 4 weeks of DMAMCL and/or ruxolitinib treatment (n = 4–7 per group). (E) RBC, ARC, and WBC in PB of chimeric recipient mice were measured following 4 weeks of treatment (n = 4–7 per group). (F and G) The *JAK2*^*V617F*^ chimera, as assessed by proportions of mutant hematopoietic cells to total cells in PB, BM (F), and spleen (G) were determined after 4 wks of treatment (n = 4–7 per group). Data are presented as the mean ± SEM. *P < .05, **P < .01, ***P < .001, ****P < .0001. ARC = absolute reticulocyte count, BMNC = bone marrow nucleated cell, CFC = colony-forming cell, DMAMCL = dimethylaminomicheliolide, MCL = micheliolide, ns = not significant, RBC = Red blood cell, WBC = white blood cell.

MPN arises from HSPCs harboring driver mutations.^30^ To determine whether MCL can target disease-causing *Jak2*V617F-mutant HSPCs, we first explored the effects of MCL on murine *Jak2*V617F hematopoietic progenitors in vitro. C-kit^+^ cells from *Jak2*^*V617F/+*^/tdTomato/*Vav1*-Cre (*Jak2*^*VF*^*/td*) and *Jak2*^*WT*^ mice were mixed in equal proportions and then cultured with MCL and/or ruxolitinib. Treatment with MCL alone (5 μM) or in combination with ruxolitinib for 48 hours significantly reduced the proportion of *Jak2*V617F-mutant cells (*P* < .05); in contrast, ruxolitinib alone (50 or 100 nM) did not change the percentage of mutant cells (Fig. 4A). In addition, BMNCs from *Jak2*^*VF*^*/td* and *Jak2*^*WT*^ mice were mixed at a 1:1 ratio and seeded in methylcellulose with MCL and/or ruxolitinib for 7 days. Numbers of *Jak2*V617F-mutant colonies were considerably decreased in response to MCL, and the addition of ruxolitinib enhanced this effect (*P* < .0001, Fig. 4B). More importantly, MCL significantly decreased the proportion of *Jak2*V617F-mutant colonies compared with vehicle treatment (25.4% ± 3.2% vs 52.5% ± 3.5%, *P* < .0001). Ruxolitinib alone was insufficient to alter the percentage of mutant colonies (48.5% ± 4.9% vs 52.5% ± 3.5%), although it increased the effect of MCL (17.5% ± 4.2% vs 25.4% ± 3.2%, *P* < .0001) (Fig. 4B). These results indicate that MCL preferentially inhibits *Jak2*V617F-mutant hematopoietic progenitors ex vivo.

Next, we generated a chimeric mouse model by transplanting BM cells from both *Jak2*^*VF*^*/td* and *Jak2*^*WT*^ mice to evaluate the potency of DMAMCL in reducing the *Jak2*V617F allele burden in vivo (Fig. 4C). DMAMCL obviously reduced red blood cell and white blood cell counts, as well as the spleen weight of chimeric mice (*P* < .05); combination of DMAMCL with ruxolitinib significantly amplified ruxolitinib-driven responses, as indicated by greater decreases in ARC (*P* < .05), white blood cell count (*P* < .05), and splenomegaly (*P* < .0001) (Fig. 4D and E). Notably, *Jak2*V617F-mutant hematopoiesis (reflected by the percentage of tdTomato^+^ hematopoietic cells in PB and BM) was clearly reduced by treatment with DMAMCL alone or combined with ruxolitinib (*P* < .05), but unaltered by ruxolitinib (Fig. 4F). The proportion of *Jak2*V617F-mutant cells in spleen was decreased by DMAMCL and combination therapy, and also by ruxolitinib (*P* < .05, Fig. 4G), possibly because ruxolitinib reduces spleen hematopoietic cell infiltration. Together, these results demonstrate that DMAMCL, but not ruxolitinib, can help reduce *Jak2*V617F-mutant allele burden in vivo.

### 3.5. MCL suppresses STAT3/5 phosphorylation by directly binding to STAT3/5 proteins

**Figure 5.**
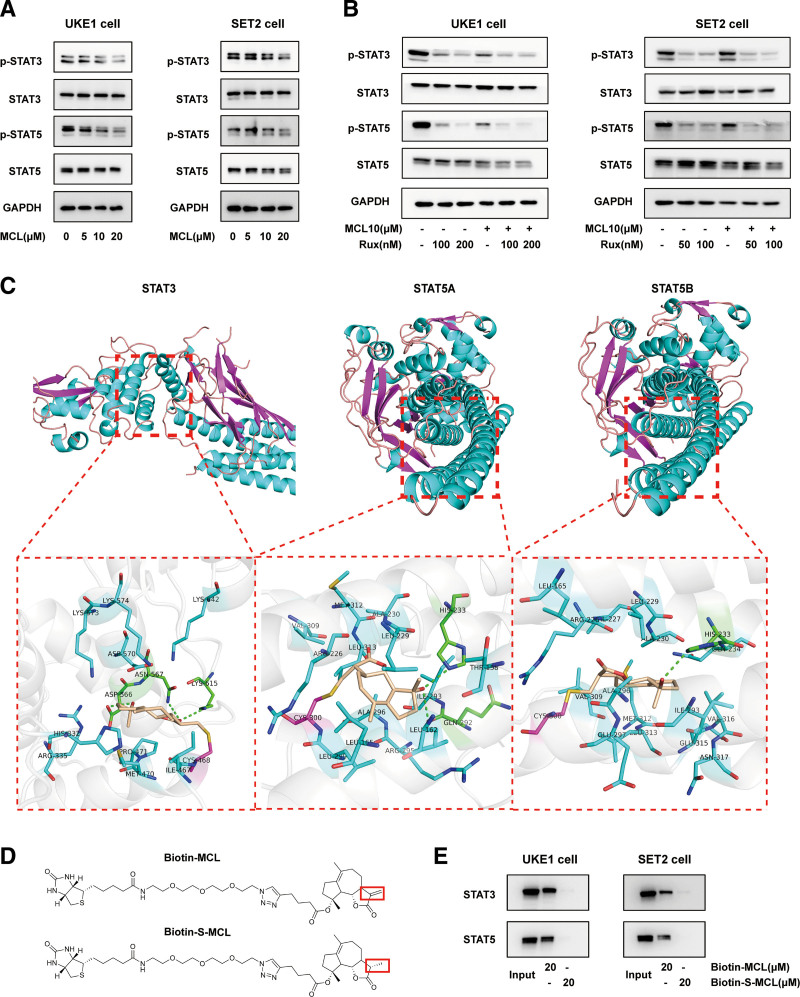
MCL suppresses STAT3/5 phosphorylation by directly binding to STAT3/5 proteins. (A and B) WB analysis was used to detect phosphorylated STAT3 (p-STAT3), STAT3, phosphorylated STAT5 (p-STAT5), and STAT5 proteins from UKE1 and SET2 cells following dose-escalated MCL (5–20 μM) (A) and MCL (10 μM) combined with the indicated concentrations of ruxolitinib (B) for 3 h. (C) The binding site and binding mode between MCL and its target proteins, STAT3 and STAT5A/B, were analyzed by AutoDock platform. Red-dot-bordered boxes in the schematics of each three-dimensional (3D) protein structure represent the binding pockets (upper). A 3D schematic of the binding pattern of MCL with STAT3, STAT5A, and STAT5B proteins is shown (lower), with interacting residues shown in blue and green, and MCL shown in a light brown carbon stick model. (D) Structure of biotin-MCL (active) and biotin-S-MCL (inactive). Red boxes represent the active 11,13-double bond (upper) and inactive reduced single bond (lower) in α-methylene-γ-lactone. (E) Cell lysates from UKE1 and SET2 cells were incubated with 20 μM biotin-MCL or biotin-S-MCL, and MCL-bound complex was separated with streptavidin agarose beads. Pull-down proteins were identified by WB with primary antibodies against STAT3 and STAT5. MCL = micheliolide, WB = western blot.

Activated JAK/STAT signaling (especially phosphorylation of STAT3 and STAT5, which activates downstream effector genes to promote cell survival and inflammatory responses) is a characteristic feature of MPNs.^31^ To gain insights into the mechanisms by which MCL affects MPNs, we performed WB analysis to determine whether MCL can suppress STAT3/5 phosphorylation. MCL elicited a dose-dependent reduction of phosphorylated STAT3/5 in UKE1 and SET2 cells (Fig. 5A). In addition, combined treatment enhanced inhibitory effects of ruxolitinib alone on STAT3/5 phosphorylation (Fig. 5B).

Previous studies^24,32^ demonstrated that the α-methylene-γ-lactone moiety in MCL reacts by Michael-type addition with biological nucleophils, the most reactive of which are sulfhydryl-containing cysteine residues in proteins, to form stable adducts and inhibit activation of target proteins. To further investigate whether there is a direct binding site between MCL and STAT3/5, we performed molecular docking analysis using the AutoDock platform. As shown in Figure 5C and Supplementary Figure 5, http://links.lww.com/BS/A73, the methylene in the α-methylene-γ-lactone ring structure of MCL forms a stable covalent bond with the sulfhydryl-containing Cys468 of STAT3 with a binding energy of −6.225 kcal/mol. Likewise, MCL covalently binds to Cys300 of STAT5A and STAT5B through the methylene with binding energies of −9.759 and −8.848 kcal/mol, respectively. In addition, MCL non-covalently interacts with multiple residues of STAT3/5, further increasing the affinity of MCL and these proteins. For instance, MCL forms three groups of hydrogen bonds with Asp566, Asn567, and Lys615 in STAT3; two hydrogen bonds with His233 and Gln292 in STAT5A; and one hydrogen bond with His233 in STAT5B. Other types of interactions observed include hydrophobic, polar, and electrostatic interactions (Fig. 5C, Supplementary Figure 5, http://links.lww.com/BS/A73).

Next, affinity pull-down experiments were carried out to confirm binding of MCL to STAT3/5 proteins. Biotin-conjugated MCL (biotin-MCL) and its inactive analogue S-MCL, in which the crucial 11,13-double bond in the α-methylene-γ-lactone moiety is reduced to a single bond (biotin-S-MCL), were prepared as positive and negative probes, respectively^24^ (Fig. 5D). After incubating UKE1 and SET2 cell lysates with biotin-MCL or biotin-S-MCL, the mixtures were separately pulled down with streptavidin-coated agarose beads and STAT3/5 was detected by WB analysis. We observed the presence of both STAT3 and STAT5 protein bands linked by biotin-MCL but not biotin-S-MCL (Fig. 5E), indicating that MCL could directly bind STAT3 and STAT5 via its 11,13-double bond in the α-methylene-γ-lactone moiety to subsequently suppress their phosphorylation.

## 4. DISCUSSION

The discovery of aberrant JAK/STAT signaling in MPNs led to the clinical use of JAK inhibitors becoming a standard treatment for intermediate- and high-risk MF and refractory PV.^4^ The JAK1/2 inhibitor ruxolitinib has become a centerpiece of MPN treatment over the last decade. Although ruxolitinib can improve splenomegaly and constitutional symptoms, and confers a survival advantage in patients with MPNs,^5,6,33^ it has some noticeable limitations. In COMFORT-I and COMFORT-II trials, approximately 50% of patients discontinued ruxolitinib treatment by 3 years, mostly because of drug-related cytopenias, suboptimal response, or disease progression^34,35^; discontinuation rates were even higher in other studies.^36,37^ Additionally, ruxolitinib has a limited effect on eliminating MPN clones, making it difficult to achieve molecular remission and cure.^9^ Many cell-intrinsic and -extrinsic mechanisms have been implicated in the inefficacy/persistence of ruxolitinib in MPNs, such as cross-talk between JAK/STAT signaling and other pathways including PI3K/AKT/mTOR, MAPK, and NF-κB^38^; JAK1/3 or TYK2 activation to rescue the effect of ruxolitinib^39^; and persistent pro-inflammatory BM microenvironment.^40^ Given that ruxolitinib monotherapy is not a remission-inducing therapeutic approach, there has been much interest in developing ruxolitinib-based rational combinations to counteract cytopenia and obtain deeper responses, such as a PI3K inhibitor^13^ and BET inhibitor that targets NF-κB.^41^

Sesquiterpene lactones (SLs) are a group of natural bioactive compounds derived from plants.^42^ SLs exert pharmacological effects mainly through a specific moiety, α-methylene-γ-lactone, which can connect to the nucleophilic structures of target molecules through Michael’s reaction, especially the sulfhydryl-containing cysteines of proteins.^32^ MCL is an SL with demonstrated efficacy for multiple malignancies.^17,18,43^ Its pro-drug, DMAMCL, which constantly releases MCL in vivo, has remarkable efficacy as a cancer therapy in a safe dose range. However, the therapeutic potential of MCL in MPNs remains undetermined. In this study, we found that MCL significantly inhibited colony formation of HSPCs derived from patients with MPNs, as well as the viability of *JAK2*V617F-mutated MPN cell lines. Moreover, MCL can enhance the effect of ruxolitinib. In vivo experiments show that DMAMCL considerably improves the efficacy of ruxolitinib in reducing splenomegaly and cytokine production in *Jak2*^*VF*^ mice without systemic or hematological toxicity. Importantly, MCL/DMAMCL preferentially targeted *Jak2*V617F-mutated cell lines and primary HSPCs, thus reducing disease burden in vivo. Taken together, our preclinical data suggest that combination therapy of MCL with ruxolitinib may provide greater benefits in MPN settings.

MCL has multiple biological activities. NF-κB is a well-recognized target of MCL whose activity is greatly inhibited by MCL.^14–16^ In addition, MCL was reported to elevate intracellular reactive oxygen species to induce apoptosis of malignant cells.^18,44^ NF-κB, which is systemically activated in MPNs, is a key transcriptional factor that promotes inflammatory responses and is required for maintenance of tumor stem cells.^41,45^ Accordingly, NF-κB may be correlated with the observed reduction of cytokines and *JAK2*V617F mutant allele burden caused by MCL in *Jak2* mice. However, apart from these known targets, other essential mechanisms by which MCL exerts effects in MPNs remain unclear.

Hyper-activated JAK/STAT signaling is fundamental to MPN pathogenesis. Phosphorylated and subsequently dimerized STAT family members bind to DNA and transcriptionally activate downstream target genes involved in oncogenic processes and inflammatory responses. Among them, STAT5 is the most critical regulator of cell proliferation and survival,^46,47^ while STAT3 is essential for pro-inflammatory cytokine production; indeed, pan-hematopoietic STAT3 deletion remarkably attenuated cytokine secretion in an MPN mouse model.^48^ Here, we observed dose-dependent inhibition of both STAT3 and STAT5 phosphorylation following MCL treatment, which probably plays an important role in the anti-inflammatory and anti-tumor effects elicited by MCL. In addition, by performing molecular docking and affinity pull-down experiments, we found that MCL inhibited STAT3/5 activity mainly through stable covalent binding to sulfhydryl-containing cysteines of these proteins, consistent with the previously reported modification pattern by which MCL regulates other target proteins.^24,32,49^ The precise cysteine residues bound by MCL require further experiments to verify. Inhibition of STAT3/5 activity by MCL with a distinct contribution from ruxolitinib, enhances blockage of JAK/STAT signaling in MPNs, thereby improving the therapeutic efficacies of ruxolitinib (including reducing splenomegaly and serum cytokines). Intriguingly, patients with greater levels of STAT3 phosphorylation inhibition are reportedly more likely to achieve a spleen response,^50^ consistent with the greater spleen volume reduction we observed in mice following combined therapy.

## 5. CONCLUSIONS

In summary, the present study shows that MCL is an effective agent against MPNs because it can covalently bind to STAT3/5 proteins to inhibit JAK/STAT signaling. Given its capability to enhance the efficacy of ruxolitinib in reducing splenomegaly, especially by targeting Jak2V617F-mutant cells to yield molecular responses, MCL may be a promising drug in combination with ruxolitinib. This finding may particularly benefit patients who have a suboptimal response to ruxolitinib, especially those with unsatisfactory spleen volume reduction. However, our preclinical results require further validation in clinical studies.

## ACKNOWLEDGMENTS

Supported in part by the Haihe Laboratory of Cell Ecosystem Innovation Fund (22HHXBSS00033), CAMS Initiative Fund for Medical Sciences (Nos. 2022-I2M-1-022), Clinical Research Fund of National Clinical Research Centre for Blood Diseases (Nos. 2023NCRCA0117 and 2023NCRCA0103), and National Natural Science Funds (Nos. 82170139, 82104785, 82070134 and 81530008).

## AUTHOR CONTRIBUTIONS

Z.J.X., Y.C., G.H., L.W., and H.J.H. conceived the idea; Z.J.X., H.J.H., and J.Q.L. designed the research; H.J.H., J.Q.L., L.Y., Y.R.Y., and M.C. performed research; B.L., Z.F.X., T.J.Q., and S.Q.Q. collected clinical data; H.J.H. and J.Q.L. analyzed data and performed statistic and bioinformatic analyses; H.J.H. and Z.J.X. wrote the manuscript; and all authors reviewed and approved the manuscript.

## Supplementary Material


