# Supplementary material for: Micheliolide exerts effects in myeloproliferative neoplasms through inhibiting STAT3/5 phosphorylation via covalent binding to STAT3/5 proteins
Source: Blood Sci. 2023 Jul 12;5(4):258–68. doi: 10.1097/BS9.0000000000000168 (PMC10629731; doi:10.1097/BS9.0000000000000168)
Supplement: Supplementary file 1 [file bs9-5-258-s001.pdf]

Supplementary Methods and Tables

Supplementary Methods

Blood and tissue histological analyses

Peripheral blood (PB) was collected from the retro-orbital plexus and measured by an automated counter (SYSMEX, XN-1000 V). Spleens were weighed, and then were fixed in 10% neutral-buffered formalin for 24 h and embedded in paraffin. Paraffin blocks were sectioned at 3 μm and stained with hematoxylin and eosin (H&E) (Solarbio) according to the manufacturer's instructions.

Flow cytometry

Murine BM cells were collected by flushing both femurs and tibias. Spleen cells were prepared by crushing and passing the tissue through a 70-μm cell strainer (BD Biosciences). PB was collected from the retro-orbital plexus. BM, spleen and PB nucleated cells were isolated by lysing in red blood cell (RBC) lysis buffer (Solarbio). Single-cell suspensions were prepared and stained with antibodies in 2% fetal bovine serum (FBS, Gibco) /PBS for 30 min at 4°C after removal of RBCs, except for erythroid precursors analysis stained before RBCs lysis. The following antibodies were used: Lineage Antibody Cocktail (145-2C11, RB6-8C5, RA3-6B2, Ter-119, M1/70; Biolegend), CD117 (c-kit, 2B8; BD), Ly-6A/E (Sca-1, D7; BD), CD34 (HM34; Biolegend), CD16/CD32 (FcγRII/III, 2.4G2; BD), CD48 (HM48-1; Biolegend), CD150 (SLAM, TC15-12F12.2;

Biolegend), CD45R/B220 (RA3-6B2; BD), CD3 (17A2; BD), Ly6C and Ly6G (Gr-1, RB6-8C5; BD), CD11b (M1/70; BD), CD71 (RI7217; Biolegend), TER-119 (TER-119; BD). DAPI (Beyotime) was added to exclude dead cells before measurement on a FACS Canto II flow cytometer (BD). Jak2V617F allele burden was determined as fraction of TdTomato-positive cells in PB, BM and spleen cells.

Cytokine measurement

Levels of cytokines in murine serum were measured by MesoScale Discovery (MSD) assay based on electrochemiluminescence technology. A total of 10 cytokines were detected: TNF-α, IL1-β, IL-2, IL-4, IL-5, IL-6, IL-10, IL-12p70, KC/CXCL1 and IFN-γ. This assay was performed by Univ biotech Co (China).

Molecular docking

The potential active sites in proteins for ligand binding were determined by Cavityplus platform (1). The grid box was generated based on the predicted active pocket, with the coordinates of grid center as (31.688, 22.816, 78.475), (-16.028, 71.423, 66.070), (-15.850, 71.571, 65.094) for STAT3, STAT5A and STAT5B, respectively. The grid point number in X, Y, Z directions was set to 80×80×80, and the number of docking times was set to 100.

Since there may be unreasonable atomic contacts in the spatial structure after docking,

the energy optimization method with the Amber 14 force field (2) was employed to release these forces, thus making the conformation more stable. Briefly, the optimization process was carried out in two steps: the 1000-step "Steepest Descent Method" was performed first, and then the 500-step "Conjugate Gradient Method" was used to further optimize the structure, and the final conformation was used as a model for subsequent analysis.

Reference

1. Yuan Y, Pei J, and Lai L. Binding site detection and druggability prediction of protein targets for structure-based drug design. *Curr Pharm Des* 2013;19:2326-33.
2. Maier JA, Martinez C, Kasavajhala K, Wickstrom L, Hauser KE, and Simmerling C. ff14SB: Improving the Accuracy of Protein Side Chain and Backbone Parameters from ff99SB. *J Chem Theory Comput* 2015;11:3696-713.

Supplementary Tables

Supplementary Table 1. Clinical and molecular characteristics of MPN patients.

Pts	Gender	Age	Diagnosis	Driver mutation	VAF	Other mutations
1	Male	62	PV	JAK2V617F	57.4%	-
2	Male	25	PV	JAK2V617F	19.7%	-
3	Male	57	PV	JAK2V617F	91.4%	TET2/TP53
4	Female	31	PV	JAK2V617F	19.3%	TET2
5	Female	32	ET	CALR p.L367Tfs*46	6.3%	-
6	Male	53	ET	CALR p.L367Tfs*46	9.1%	DNMT3A
7	Female	35	PMF	JAK2V617F	39.2%	IDH2

VAF: Variant allele fraction

Supplementary Table 2. The half maximal inhibitory concentration (IC50) of MCL and ruxolitinib in UEK1 and SET2 cells respectively.

Drugs	IC50±SD	
	UKE1	SET2
MCL (μM)	12.72±0.38	9.08±1.03
Ruxolitinib (nM)	568.9±48.20	34.20±0.29

The values were calculated from three independent replicate experiments.

Supplementary Table 3. Combination index (CI) of MCL and ruxolitinib in UKE1 and SET2 cells.

UKE1 cells			SET2 cells		
MCL (μM)	Ruxolitinib (nM)	CI value	MCL (μM)	Ruxolitinib (nM)	CI value
2	100	0.76	2	6	0.99
4	200	0.89	4	12	1.12
6	300	1.04	6	18	0.84
8	400	1.13	8	24	0.88
10	500	1.29	10	30	0.98
