# Supplementary material for: Micheliolide exerts effects in myeloproliferative neoplasms through inhibiting STAT3/5 phosphorylation via covalent binding to STAT3/5 proteins
Source: Blood Sci. 2023 Jul 12;5(4):258–68. doi: 10.1097/BS9.0000000000000168 (PMC10629731; doi:10.1097/BS9.0000000000000168)

Supplementary Figure 1. The effect of MCL on cell cycle progression of MPN cells

(A) Representative flow cytometric plots of cell cycle detection and
(B) proportions of cell cycle phases of UKE1 and SET2 cells receiving
dose-escalated MCL (2.5-10 μM) treatments for 24 h.
The experiments were performed in triplicate independently.
Data are presented as the mean ± SD. * $P < 0.05$.

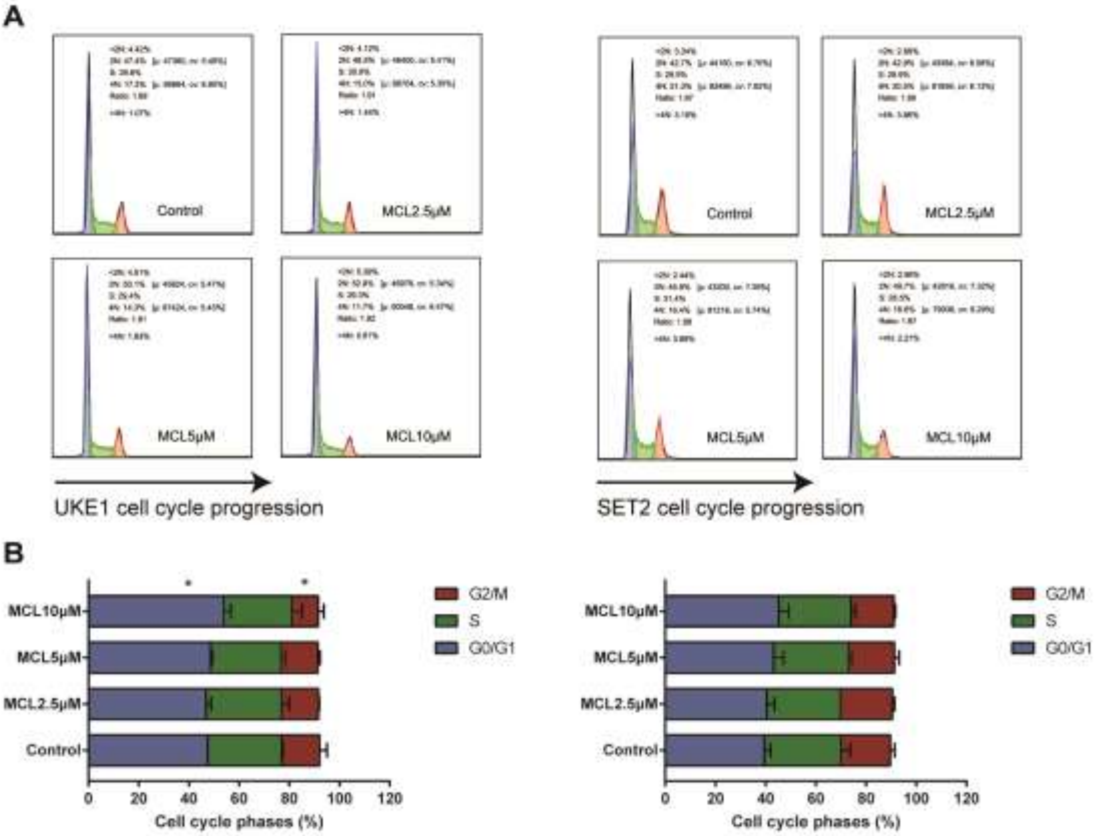

Supplement: Supplementary file 2 [file bs9-5-258-s002.pdf]
