# Supplementary material for: Micheliolide exerts effects in myeloproliferative neoplasms through inhibiting STAT3/5 phosphorylation via covalent binding to STAT3/5 proteins
Source: Blood Sci. 2023 Jul 12;5(4):258–68. doi: 10.1097/BS9.0000000000000168 (PMC10629731; doi:10.1097/BS9.0000000000000168)

Supplementary Figure 2. The JAK2V617F mutated murine cells (Ba/F3-EPOR-JAK2V617F) were exposed to varying concentrations of MCL, and the proportion of apoptotic cells were measured at 48 h. The wild-type Ba/F3 cells were used as controls. ** $P < 0.01$, **** $P < 0.0001$.

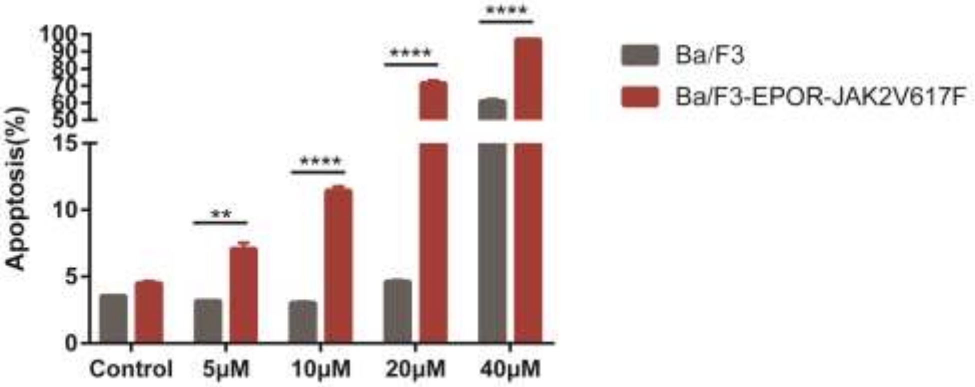

Supplement: Supplementary file 3 [file bs9-5-258-s003.pdf]
