# Supplementary material for: Micheliolide exerts effects in myeloproliferative neoplasms through inhibiting STAT3/5 phosphorylation via covalent binding to STAT3/5 proteins
Source: Blood Sci. 2023 Jul 12;5(4):258–68. doi: 10.1097/BS9.0000000000000168 (PMC10629731; doi:10.1097/BS9.0000000000000168)

Supplementary Figure 3. The toxicity assessment of DMAMCL in vivo

(A) The wild-type (WT) littermates receiving PBS or DMAMCL treatments were weighted at week 0,1,2,3 and 4 after beginning of administration (n=7 per group).
(B) Spleen and liver weight of WT mice after four weeks of PBS or DMAMCL treatments (n=7 per group).
(C) Representative images of spleens and livers from WT mice with or without DMAMCL treatments.
(D) Representative images of H&E staining in spleen and liver biopsy specimens from mice receiving PBS or DMAMCL treatments for four weeks. Original magnification 20×; scale bar 100 μm.
(E) Peripheral blood cell counts of WT mice were assessed after four weeks of PBS or DMAMCL treatments (n=7 per group).
(F-G) The proportion of HSPCs and differentiated erythroid and myeloid cells in bone marrow (BM) (F) and spleen (G) of WT mice receiving PBS or DMAMCL treatments (n=7 per group).
I, II, III, IV represent erythroid cells at different differentiative stages.

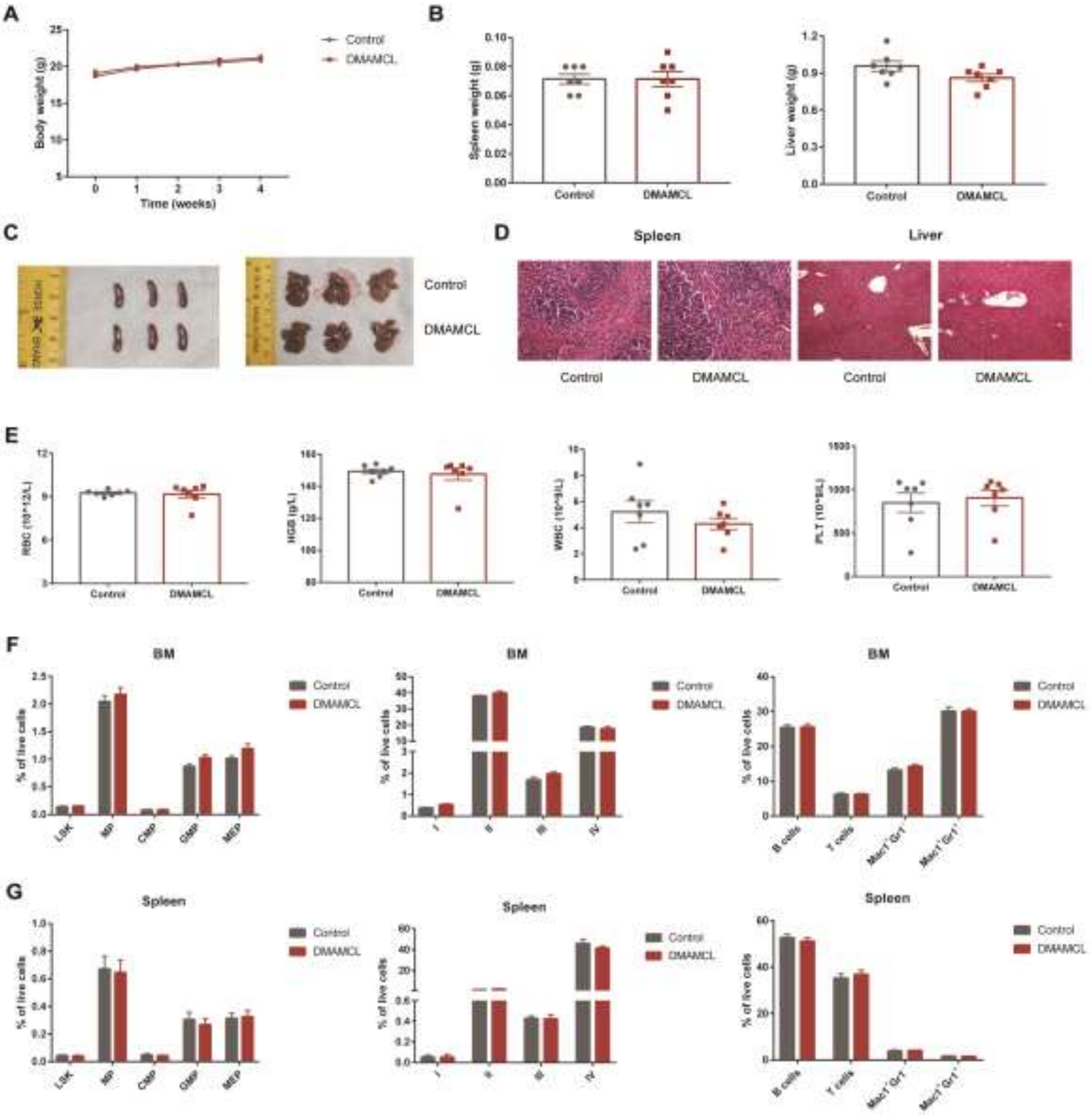

Supplement: Supplementary file 4 [file bs9-5-258-s004.pdf]
