# Supplementary material for: Micheliolide exerts effects in myeloproliferative neoplasms through inhibiting STAT3/5 phosphorylation via covalent binding to STAT3/5 proteins
Source: Blood Sci. 2023 Jul 12;5(4):258–68. doi: 10.1097/BS9.0000000000000168 (PMC10629731; doi:10.1097/BS9.0000000000000168)

Supplementary Figure 4. The efficacy of DMAMCL on the hematopoietic stem and progenitor cells (HSPCs) proportions in BM, and extramedullary hematopoiesis of Jak2VF mice

(A) Representative flow cytometric plots and (B) the proportions of Lin-Sca1+c-kit+ (LSK) cells, myeloid progenitors (MP), common myeloid progenitors (CMP), granulocyte/macrophage progenitors (GMP), and megakaryocyte/erythroid progenitors (MEP) in BM of recipient mice following 4 weeks of DMAMCL and/or ruxolitinib treatments (n=6-8 per group).
(C) Representative images of H&E staining in spleen biopsy specimens from mice receiving DMAMCL and/or ruxolitinib for 4 weeks. The black arrows point to megakaryocytes. Original magnification 20×; scale bar 100 μm.
Data are presented as the mean ± SEM. * $P < 0.05$, ** $P < 0.01$.

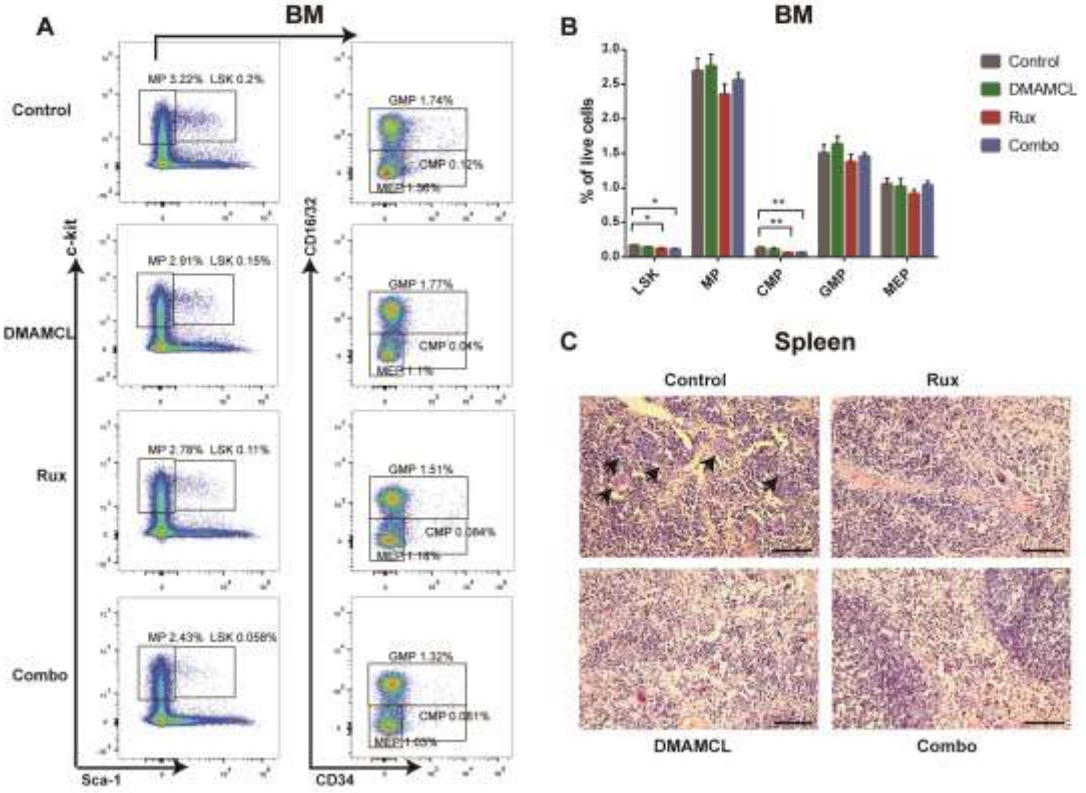

Supplement: Supplementary file 5 [file bs9-5-258-s005.pdf]
