# Supplementary material for: Micheliolide exerts effects in myeloproliferative neoplasms through inhibiting STAT3/5 phosphorylation via covalent binding to STAT3/5 proteins
Source: Blood Sci. 2023 Jul 12;5(4):258–68. doi: 10.1097/BS9.0000000000000168 (PMC10629731; doi:10.1097/BS9.0000000000000168)

**Supplementary Figure 5. The two-dimensional schematic diagram of binding sites and binding patterns between MCL and STAT3, STAT5A and STAT5B proteins.**

Black straight lines represent covalent bond, purple arrows represent hydrogen bond, green residues represent hydrophobic interaction, violet residues represent electrostatic interaction (positive), red residues represent electrostatic interaction (negative), and blue residues represent polar interaction.

The character R and S represent atomic configuration.

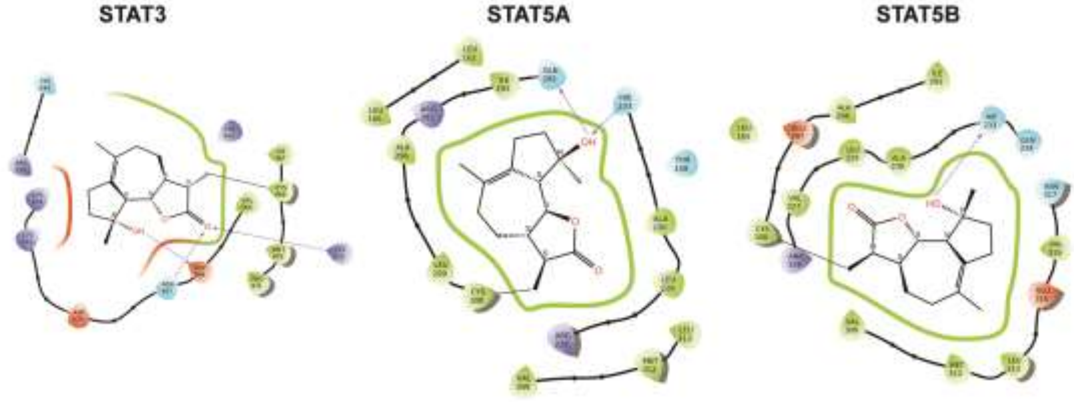

Supplement: Supplementary file 6 [file bs9-5-258-s006.pdf]
